# Organizational and social justice paradoxes in EDI

**DOI:** 10.3389/fpsyg.2024.1320993

**Published:** 2024-03-27

**Authors:** Anita Bosch

**Affiliations:** Stellenbosch Business School, Stellenbosch University, Bellville, South Africa

**Keywords:** social value, social justice, equality, need, deservingness, workplace justice, equality diversity and inclusion

## Abstract

This perspective article positions social justice as an addition to the aims of organizational justice, and core to diversity, equality, and inclusion (DEI). It problematizes simplistic DEI rhetoric and positions paradoxes within DEI, as experienced by employers, based on an explanation of key justice concepts and the introduction of fairness, equality, desert, and need. The paper broadens perspective-taking beyond a sole focus on beneficiaries of DEI, towards tensions that employers experience in working towards the aims of workplace justice, including the embeddedness of social justice within both organizations and social systems. The paper concludes with avenues for future research and a call to carefully examine simplistic notions of organizational justice in effecting DEI, suggesting a paradoxical lens on embracing, rather than avoiding, multiple and often conflicting workplace justice imperatives.

## 1 Introduction

Justice in workplace decision making is the crafting of policies, including on the allocation of resources, to ensure fair decisions by organizational leaders (Virtanen and Elovainio, 2018: 306). Reasons for the need for justice at work, also referred to as ‘organizational justice’, revolve around employees’ economic and social interests, with less attention paid to moral convictions (Cropanzano and Stein, 2009: 201). It is with the latter that social justice is concerned. Specifically, social justice is aimed at “a person or category of persons [who] enjoy fewer advantages than that person or group of persons ought to enjoy (or bears more of the burdens than they ought to bear), given how other members of the society in question are faring” (Miller, 1999: 15). Workplace justice is therefore concerned with employees’ self-oriented interests around fairness, such as minimizing outcomes like unfavorable economic results, reduced status, and a lack of control (Cropanzano and Stein, 2009: 201), and with contributing towards the balancing or correcting of advantages and burdens that may accrue disproportionately to members or groups in a particular society, which is a moral imperative. As such, justice lies at the heart of workplace diversity, equality, and inclusion (DEI), as DEI is founded on the eradication of discrimination and the building of workplace social solidarity through fairness.

However, workplace DEI interventions often suffer from fashionable rhetoric (Oswick and Noon, 2014: 36), and stop short of equipping both leaders and followers with an understanding of the complex and conflicting nature of attaining justice at work. This oversight can be attributed to managers’ linear and simplistic arguments that support rapid decision making and the avoidance of paradoxical ideas that are regarded as burdensome to unravel, or are rejected out of hand as counter to progress. Here, an example of fashionable DEI workplace rhetoric is an argument with which to increase political gains, with little consideration of feasibility (Miller, 1999: 11) and societal realities, such as simplistic notions of ‘embracing people that are different to you, else you are bigoted’ without the necessary attention to clashing values, beliefs, and cultures, or that “removing power and privilege” is the primary aim of DEI (Kohl, 2022: 5). In a similar way, managers can also use fashionable rhetoric in arguments of purported unfeasibility, towards political gains, by maintaining the status quo. An example is leaders arguing that women should not enter male-dominated jobs because they simply do not belong there.

DEI in organizations is concerned with both fairness and morality. It is morally positioned on universal human rights, which, ironically, may provide a vehicle towards perceived righteousness through fashionable rhetoric — such as taking a stance that those who do not embrace all forms of diversity and equality are ignorant, “greedy and oppressive while proponents [of diversity and equality] are compassionate” (Guerrière, 2019: 26). In addition, Western workplace DEI assumptions may show less concern for the realities and readiness for change in local workplace settings, resulting in a fairness deficit.

I argue that elucidation of the inherent paradoxes within DEI as experienced by employers may result in less rhetoric and more thoughtful approaches towards attaining the ideals of workplace justice. Thus far, I have described the overlap between organizational justice, social justice, and DEI. In addition, the purpose of this paper is to present the mechanisms of social justice that are at the disposal of employers when making DEI decisions, and to present DEI paradoxes as these relate to justice at work. The paper concludes with avenues for future research.

## 2 Organizational and social justice

Justice is the creation and application of rules based on what is morally right (Furby, 1986: 188). It therefore stands to reason that the rules of justice may be fallacious if their moral underpinning is misguided or without substance. For instance, prior to democracy, South African laws mandated apartheid, a policy of exclusion based on race, which was immoral and, therefore, unjust. Workplace organizational justice is underpinned by three types of justice, namely *distributive justice* (the justice of the outcomes of distribution decisions), *procedural justice* (justice of the procedure of the formal allocation of resources), and *interactional justice* (the justice of interpersonal transactions between people and groups) (Cropanzano et al., 2007: 36). Viewing social justice as part of the ambit of organizational justice, I also include the balancing of the societal advantages and burdens attached to individuals and groups in workplace justice concerns. Whereas social justice may have previously been viewed as solely the responsibility of governments and welfare agencies commissioning measures such as education and poverty alleviation, paid work and employment are powerful means by which societal burdens attached to individuals and groups can be distributed differently. This, in addition to demands for fairness from employees, employers are also squarely tasked with the moral imperative of social justice since workplaces interface with receiving labour from disenfranchised groups and individuals. Workplaces, for instance, are not tasked with social justice as it relates to children directly — unless employers hire children or produce products aimed at children. However, for purposes of this paper, the interface between organizational and social justice relates to individuals and groups of working age who offer skills and labour towards the attainment of organizational outcomes.

## 3 Fairness and justice

In contrast to justice, fairness relates to “impartial treatment, without favoritism or discrimination” in “the absence of significant differences” between people or cases (Furby, 1986: 155; Kolosko, 2014). Workplace DEI invites difference, not only in group affiliation such as different genders, races, ethnic origin, and classes, but also in individual differences such as cognitive functioning, skill sets, and personality. It follows that there are differences between people at work in any organization, and more so in organizations that deliberately seek out difference. Impartial treatment, which would seemingly lead to fairness, may therefore become complicated for employers. For instance, employees use their levels of relative deprivation to determine the fairness of distributions they receive (Stouffer et al., 1949 in Virtanen and Elovainio, 2018: 306); they do not base their impressions of fairness on absolute levels, but rather on how they regard a comparison of their “rewards with those of others” (Virtanen and Elovainio, 2018: 306). If they feel deprived relative to what they perceive others have, they will consider the situation or outcome unfair. Judgements of fairness are therefore subjective, and fairness is not the same as justice (Goldman and Cropanzano, 2015: 317), as the latter relates to morally informed rules, irrespective of perceptions of fairness.

There are various avenues in understanding how employees judge the fairness of the actions of employers. For instance, in order to be considered fair, components of *procedural justice* relate to consistency in treatment, accuracy of information, representation of all, an absence of bias towards groups or individuals, and the ability to correct errors when discovered (Cropanzano et al., 2007: 36). *Interactional justice* is about preserving the relationship though “dignity, courtesy and respect” and sharing relevant information with employees (Cropanzano et al., 2007: 36). Both procedural and interactional justice hold important implications for leaders’ justice decision making and the eventual perception thereof as being fair. It is, however, *distributive justice* that may aid leaders in taking complex decisions, especially as it relates to the social justice and DEI imperative, since social justice is concerned with the balancing (or distributing) of advantages and burdens disproportionately allotted to individuals and groups in society.

## 4 Principles of social justice at work

For employers, distributive justice relies on leaders creating and applying workplace rules and procedures that balance “claims and counter-claims … in a procedure designed to avoid destructive conflict” (Hampshire, 1989: 63). Distributive justice deals with justice decisions where “not all workers are treated alike” (Cropanzano et al., 2007: 38), and the aim is not necessarily to treat all workers in exactly the same manner. Miller (1999) suggests that there are three social justice principles at play when weighing distributive justice, namely desert (merit), equality, and need.

### 4.1 Desert

Desert or deservingness “is typically limited to situations involving merit” (Furby, 1986: 188), whereby employees receive rewards according to their contributions (Cropanzano et al., 2007: 36). From an organizational justice perspective, employers that apply criteria of merit reward individual excellence, which leads to perceptions of fairness. However, merit is a concept in DEI that is fraught with contestation (Vijay and Nair, 2022: 315), mainly regarding the assumptions that underpin criteria to determine merit. Leaders may evaluate outcomes by applying historical criteria without examining structural historical — often invisible — inequalities inherent in workplaces. Such historical criteria develop over time based on assumptions about the capabilities of employees present in workplaces, without regard for differences in the life journeys and daily realities that have an impact on their capabilities. Individuals who wish to enter or have entered workplaces and have different lived experiences to those who were traditionally in those spaces may agitate for an adjustment of the criteria used to determine merit or desert, with the aim of establishing fairness. Merit then contains a social justice imperative. It is not only about a favored group or individuals who have enjoyed a specific life journey leading to outcomes in line with the benefits of that journey, but also about an acknowledgement and taking into account differences in life journeys, as these differences may lead to barriers to reaching outcomes that are, under existing criteria, regarded as meritorious. Social justice at work means that merit is no longer only about the contributions that a person makes according to pre-determined criteria and employers determining rewards at the end of a process or project. Merit, or deservingness, under social justice manifests when employees who, either individually or as part of a group, share disproportionately in societal burdens are selected for entry, for instance, to the organization’s employ or participation in a work project. When considering social justice, merit thus involves the contributions that employees have the *potential* to make in future, even though they have not yet had the opportunity to showcase these.

### 4.2 Equality

Equality is a cornerstone concept of DEI and distributive justice. Buchanan and Mathieu (1986: 15) provide guidance in stating that “inequality of treatment is not in itself unjust; what is unjust is unequal treatment for irrelevant reasons.” Paradoxically, equality is then not sameness or the exact same treatment for all. Egalitarian leaders may specifically focus on equality of outcome — the distribution of social and material goods towards equal results (Phillips, 2004: 1). Simply put, each person should end up with, roughly, the same as the rest. Equality of opportunity, on the other hand, focuses on every employee having an equal chance to succeed, which requires ‘leveling the playing field’ at the start of the game (Roemer and Trannoy, 2016: 1289). Simply put, everyone gets the same at the start, but may not end up having the same at the end. However, equality of opportunity should be tempered through the lens of *complex equality*, as “we need to situate [equality] in the context of concrete and historical relationships” (Walzer, 1983: 68) that flow from unequal social arrangements resulting in differential starting points and outcomes for individuals and groups. The concept of equity is often used to deal with matters of complex equality. Equity may be seen as based on merit “to each in accordance with their contributions” (Cropanzano et al., 2007: 37), or with an introduction of needs to address complex equality — to each in accordance with their needs.

### 4.3 Need

Employers are often perplexed about their responsibility for social justice based on needs. Needs are defined “by reference to a minimal standard of life” in a particular community (Miller, 1999: 225), and will differ between people and societies. Needs introduce differences between people based on aspects that are largely out of their control, such as illness or disability, but can also be related to group differences, such as women requiring lactation facilities at work. Depending on the resources in a society, the economic want of the poor may also be considered a need, and government intervention may therefore take the form of making the payment of a living wage mandatory (Stone and Kuperberg, 2008). In economically constrained societies, being poor is a widespread reality, dealt with through subsistence farming and other forms of survival activities, potentially making the payment of a living wage a desired future state but an inappropriate strategy for the realities of that specific society.

For purposes of employers, needs should be agreed on as a set of minimum standards that each employee can legitimately expect to have met and that an employer willingly contributes to, for instance, that all employees must have a computer and access to stable internet to enable them to perform their work, that the workplace will be safe and free of harassment, and so forth. Reflection on needs adds an important dimension to leaders’ justice decisions, and also balances decisions about equality and desert by deliberately including those who are most at risk in workplace communities when considering workplace social justice.

## 5 Paradoxes underpinning social justice at work

The paradoxes discussed below are inherent in social justice and provide a lens of complexity which enriches employer DEI considerations. The interlinkages between employer and societal concerns are evident in each of the paradoxes, pointing towards the embeddedness of DEI, which is ordinarily viewed as within the purview of an organization, within society and its mechanisms that foster justice for all.

### 5.1 Paradox of needs

The paradox in the discussion of needs relates to leaders having to make decisions about the acknowledgement of *needs as justice*, i.e., the needs that employers are morally obligated to address, such as a blind employee requiring special computer software, even though it may be costly. In contrast, there is the fulfilment of needs through *generosity and humanity* on the part of the employer (Miller, 1999:89). The choice of generosity may differ according to societal needs. In instances where the organization has plenty, the demand for meeting *needs as justice* may be higher. Societies that struggle economically may thus rely on the fulfilment of their needs by benevolent employers.

### 5.2 Paradox of social value

Societal advantages and burdens are very broad concepts, more so with respect to the responsibility of employers towards balancing them. Therefore, per society, there should be “broad consensus about the social value of a range of goods, services, and opportunities” (Miller, 1999: 22), and the value assigned should be “independent of a particular person receiving them” (Miller, 1999: 23). Broad consensus on the social value (therefore, also the importance) of social justice, brings the responsibility for social justice closer to employers. Social value has to do with “the ultimate meaning of how we are to live” (McMurtry, 2009, cited in Baruchello and Johnstone, 2011) and with human survival that is founded on collaboration (Corning, 2003). Paradoxically, there is an assumption in the domain of workplace DEI that employers subscribe to the value that society — universally — attaches to the inclusion and equality of disenfranchised individuals and groups.

### 5.3 Paradox of the productive economy

The inclusion of marginalised groups into workplaces is based on assumptions that there are sufficient openings in the job market or that the number of productive jobs or business opportunities are on the increase, and that organizations are therefore able to accommodate new entrants. Sustainable job creation is dependent on a productive economy, including healthy legislative, political, labour, natural and market forces (Wulandari et al., 2017). For instance, due to an ageing population, Europe may welcome migrant workers from diverse cultures to fill multiple job openings (Peri, 2020). However, in South Africa, where there is a very high unemployment rate, high numbers of youth, and shrinking business base (StatsSA, 2024), employers may end up replacing existing incumbents with individuals from marginalised groups, especially where legislation directs who is to be appointed, paradoxically conflating economic scarcity with social justice. The DEI ideal of a broadening participation and inclusion for all is diminished. Even though there is strong recognition for the importance of social justice imperatives in South Africa, when replacement becomes a main feature of workplace DEI efforts under conditions of economic scarcity, a balance between morality and perceptions of fairness is placed under unwarranted pressure, which may amplify perceptions of exclusion.

### 5.4 Paradox of time

The time horizons attached to workplace justice and social justice are different. Workplace justice relates to self-oriented (Cropanzano and Stein, 2009: 206) employee concerns about justice in relation to a specific employer, which is short-term in nature. Social justice, which involves an employer attending to the distribution of societal advantages and burdens to address disproportional representation, exclusion, or discrimination, has a long-term horizon. Herein lies one of the greatest paradoxes for employers — organizational or workplace justice leads to improved business outcomes in the short term, with near-immediate economic benefit to the employer. Social justice requires employers to make long-term investments that indirectly benefit the employer through social cohesion, the reduction of poverty, and an increase in share of voice — but over the long term.

## 6 Closing

The debate about fairness and justice in DEI revolves around the shifting of societal beliefs regarding what constitutes legitimate expectations (Furby, 1986: 192) and who has the power and legitimacy to interpret unfairness. Contestations are ever-present, and will continue into perpetuity. Therefore, DEI can no longer rely on simplistic notions in matters of workplace justice in the hope of doing the right thing. It is time to actively introduce complexity into workplace justice decisions by highlighting, acknowledging, and discussing paradoxes and inconsistencies. Such complexities eliminate managers’ propensity to over-simplify workplace decisions by likening equality with sameness of treatment and outcome. It also broadens perspective-taking beyond a sole focus on beneficiaries of workplace justice, towards tensions that employers experience in working towards the aims of DEI.

Acknowledging this complexity brings a fresh quest to DEI efforts — both leaders and followers have to be deliberately and thoroughly equipped to think through the various angles from which decisions can be viewed and outcomes shaped. Future research should expand on applications of equality, desert, and need within organizational DEI experiments involving distributive justice. The development of case examples could serve as useful material in developing paradoxical thinking in leaders. An exploration of approaches that achieve broad consensus about social value could add to our understanding of more closely binding employers to the responsibility for social justice. Studies involving multiple economic contexts and economic levers could be correlated with perceptions of fairness amongst employers, beneficiaries of social justice, and those whose disproportionate share in societal benefits are being reduced. Needs as justice should be distinguished from needs as benevolence, as viewed by employers, within specific economic contexts, towards clarification of the criteria that employers may use in making distinctions between needs. Furthermore, exploring theory from social justice disciplines such as philosophy and law may aid inter-disciplinary theory development and provide new insights towards improved DEI social justice outcomes. What is clear is that, in the absence of acknowledging paradoxes inherent in DEI, the runaway train of fashionable simplistic DEI rhetoric threatens to derail interventions towards just workplaces and robs us of our agency in crafting a future that we all regard as fair.

## Data availability statement

The original contributions presented in the study are included in the article/supplementary material, further inquiries can be directed to the corresponding author.

## Author contributions

AB: Conceptualization, Writing – original draft, Writing – review & editing.
